# Identification, Expression and Target Gene Analyses of MicroRNAs in *Spodoptera litura*

**DOI:** 10.1371/journal.pone.0037730

**Published:** 2012-05-25

**Authors:** Zhongchen Rao, Wenyin He, Lin Liu, Sichun Zheng, Lihua Huang, Qili Feng

**Affiliations:** Guangdong Provincial Key Lab of Biotechnology for Plant Development, School of Life Sciences, South China Normal University, Guangzhou, China; Niels Bohr Institute, Denmark

## Abstract

MicroRNAs (miRNAs) are small RNAs widely present in animals and plants and involved in post-transcriptional regulation of gene transcripts. In this study we identified and validated 58 miRNAs from an EST dataset of *Spodoptera litura* based on the computational and experimental analysis of sequence conservation and secondary structure of miRNA by comparing the miRNA sequences in the miRbase. RT-PCR was conducted to examine the expression of these miRNAs and stem-loop RT-PCR assay was performed to examine expression of 11 mature miRNAs (out of the 58 putative miRNA) that showed significant changes in different tissues and stages of the insect development. One hundred twenty eight possible target genes against the 11 miRNAs were predicted by using computational methods. Binding of one miRNA (sli-miR-928b) with the three possible target mRNAs was confirmed by Southern blotting, implying its possible function in regulation of the target genes.

## Introduction

MiRNAs are small endogenous regulatory RNAs. They usually are only about 22 nucleotides long and their precursor can fold into a stem-loop structure [1]. Since the second miRNA let-7 was reported in 2000 [2], these vital participators in post-transcriptional gene regulation have received more and more attention and many efforts have been made to discover new miRNAs in different species. More than 15000 miRNAs have been identified from different species, such as *Bombyx mori*, *Caenorhabditis elegans*, *Arabidopsis thaliana* and *Homo sapiens*, by either computational or experimental method and deposited in the miRbase (release 16.0 by mirbase) (http://www.mirbase.org).

MiRNA plays important roles in many physiological processes, such as growth, development, metabolism, behavior and apoptosis by mRNA cleavage or translational repression [1], [3], [4], [5], [6], [7], [8], [9]. It is found that one miRNA can target mRNA of several genes. In human, mRNAs of one-third of genes are regulated by miRNA as transcriptional or developmental factor; on the other hand, one molecule of mRNA can be bound by several different miRNAs [10].

Cloning usually is a direct approach to identify miRNAs, however, significant variation in their expression levels has made it difficult to clone low abundant miRNAs [11], [12]. Therefore, computational methods have become a helpful approach to identify miRNAs by searching for signature structure of miRNA molecules [13].

So far, the research of miRNA mainly focuses on mammals (such as *Homo sapiens* and *Mus musculus*) and eudicotyledons (such as *Medicago truncatula* and *Arabidopsis thaliana*). Insect miRNA identification is far behind nematodes, mammals and plants. Totally more than 2300 insect miRNAs have been identified from 22 insect species, including *Drosophila melanogaster*, *Anopheles gambiae*, *Apis mellifera*, *Bombyx mori*, *D. pseudoobscura* and deposited in miRbase (release 16.0). Most of these insect miRNA are identified by computational method and have not been experimentally validated. No miRNA are reported in agricultural pest insects.

In this study we used computational and experimental methods to identify miRNAs in the lepidopteran species *Spodoptera litura*, one of the most destructive agricultural insect pests in tropical and subtropical areas of the world. We examined the spatial and temporal expression profiles of these miRNAs in the midgut, epidermis and fat body during development from egg to adult stages. We also predicted possible target genes for some of these identified miRNAs.

## Results

### Computational Identification of *S. litura* miRNAs

**Figure 1:**
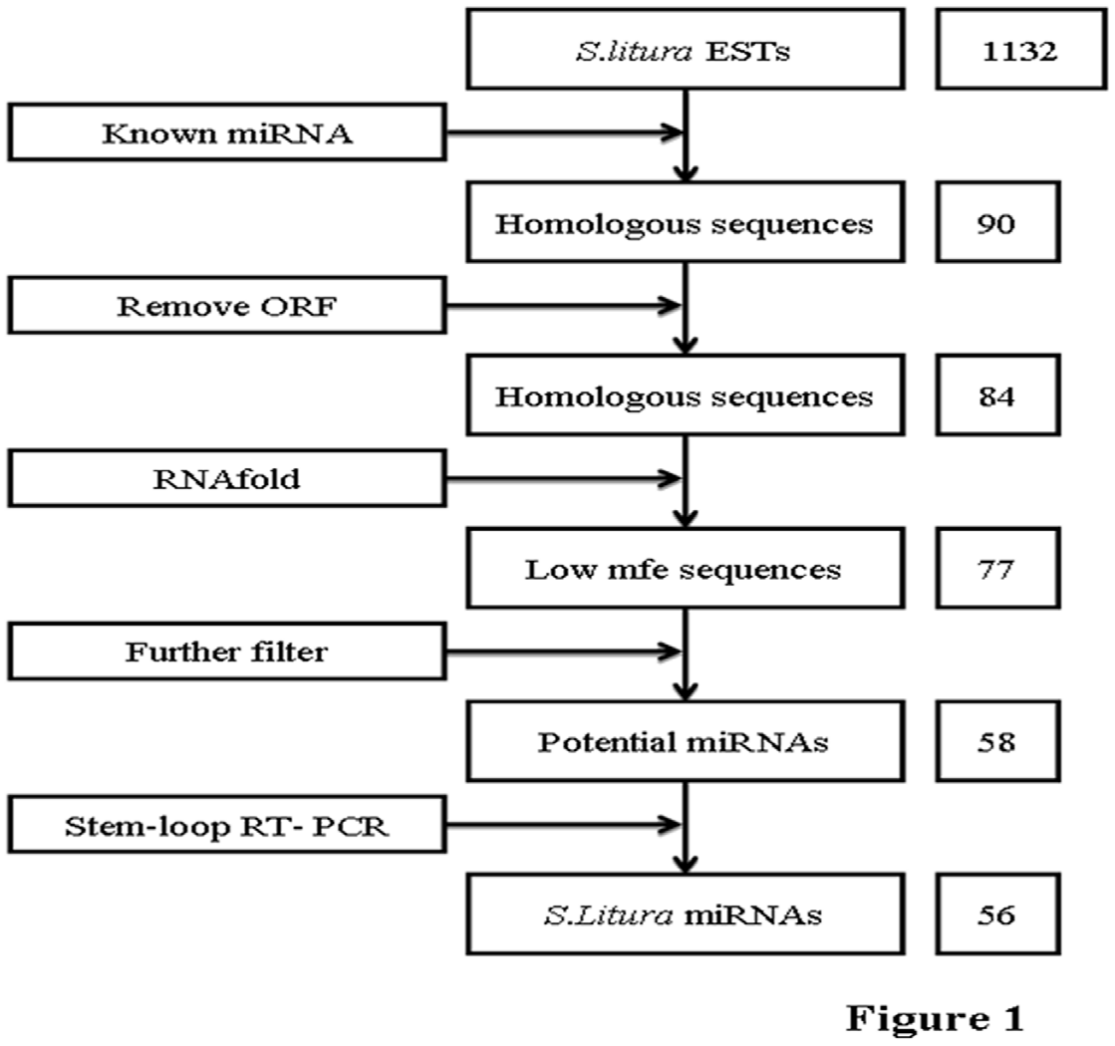
Identification pipeline of miRNA from *Spodoptera litura.* The numbers in the boxes on the right are the ones for corresponding ESTs or miRNAs identified.

Homologue search method was used to identify miRNAs in *S. litura*. By using bl2seq program to analyze 1,132 un-annotated ESTs, 90 sequences were found to contain the fragments homologous to the 403 known miRNAs from five insect species, including *B. mori*, *A. gambiae*, *D. pseudoobscura*, *D. melanogaster* and *A. mellifera* in the miRbase (release 12.0) (Fig. 1). The homologous regions of these 90 sequences had no more than five nucleotides mismatch to the known miRNAs. Approximately 100 bp fragments, including the homologous region and the upstream and downstream flanking regions, of these 90 sequences were extracted and used for RNA secondary structure analysis by RNAfold [14]. Fifty eight fragments were found to satisfy the prerequisite of a free energy threshold [15] and considered to be encoded by potential miRNAs. According the nomenclature of miRNA [16]–[17], these *S. litura* miRNAs were signed identity as shown in Table 1.

**Table 1:** Predicted miRNA in *Spodoptera litura*.

No.	miRNA	Mature sequence	No.	miRNA	Mature sequence
1	sli-miR-211	CUGUGCGUUGUACACACGGCUA	30	sli-miR-928b	GUGGCUGUAGAGGCGGCGAC
2	sli-miR-125	UCACUGAGACCAAAACUCCUGA	31	sli-miR-1890b	UGACAUAAUAACGAUUUCA
3	sli-miR-283a	UAAGUAGUAGCUUUAAUUCU	32	sli-miR-928a	CUGGCUGUGGAGCUGGCGCU
4	sli-miR-928c-1	CUGGCUGUGGAAGCUGGCGAA	33	sli-miR-970	ACAUACUACACUACCCGGCUAU
5	sli-miR-13	CACAUCACUAGGGCUGUGAUA	34	sli-miR-928c-2	CUGGCUGUGGAAGCUGGCGAA
6	sli-miR-10	ACAAAUUCGGAUCAAAAGACA	35	sli-miR-71	UGAAAGACUGGUGUGAGUGA
7	sli-miR-1890a-1	UGACAUCUUUGAUUAGGUCU	36	sli-miR-1000	ACUCCUGUCCAAGACAAUAA
8	sli-miR-286	UGAUUAGACCUACACACUCGCG	37	sli-miR-29b	GCUGAGCCCAAAUGGGGCUA
9	sli-miR-263b-1	AUUGACACCGGAAGAAUUCGC	38	sli-miR-263b-2	GUGCAUACUUCAUGCCAAG
10	sli-miR-305-1	AUUGUACUUCUUGUUGGUCUG	39	sli-miR-983	UCAUUAGAUCAUACGCACUAU
11	sli-miR-283b	UUAAUAACCACGCUAAUAUUUA	40	sli-miR-307	UCAGCAUCUCCUUGGGUGAC
12	sli-miR-283c	AAAAUGUCGCUGGUAAUUCC	41	sli-miR-1175	UUACUUCGUGAGAGUAGAAACUCA
13	sli-miR-79	AUGCUAUUUUAAAUAUAGCUUUA	42	sli-miR-316	UGUGUUUUUCACUUUGCUGCAG
14	sli-miR-954	UCUGGGUGUCAUUGUGUAU	43	sli-miR-184	UCGACGAACAACUAUAAGGG
15	sli-miR-981	UUCGUAUGACAUGAAACCUG	44	sli-miR-15	CGGACGGAGUAGUCUUUAGGG
16	sli-miR-31a	CACAAUAUGUCGCGUAGCUGA	45	sli-miR-278	GCCCAUUUGACUUGCCGUCCA
17	sli-miR-989	UGUGAUGCUAAUGUAGAUGCUAC	46	sli-miR-14	CGGGGAGAGAACUGGAAGAGG
18	sli-miR-210	UUGUUCGUGAUCGCAGCGGCUU	47	sli-miR-2771	UAACAUUAUGAGGAUGGGUUGAACUG
19	sli-miR-1006	UAAGAAUAGGAAGAACUCGAAUUU	48	sli-miR-2809	AAAACAGCAGACGGAUCACCUGAU
20	sli-miR-6	AAAAAGACCAGCCCUCUGGUA	49	sli-miR-3001	AACUCUACAAUUAUUUAAAUUUA
21	sli-miR-1890a-2	UGACAUCUUUGAUUAGGUCU	50	sli-miR-3242	CACGCGGGCGGGCGUAUCGCGUUGGA
22	sli-miR-7	UGAGAUAAAGGUUGUUUUGU	51	sli-miR-3279	UAUGUUAUACAAUAUUUAUGACU
23	sli-miR-33a	GUGCAUUUGUAGUCGCAUU	52	sli-miR-3291	AAAUUCAUAUAUUUAGGUACAAA
24	sli-miR-981	UUCGUUGUAAGAAACUUACA	53	sli-miR-3290	UGUGUGAUUAUACUUAUGAUUUU
25	sli-miR-305-2	AUUGUACUUCUUGUUGGUCUG	54	sli-miR-3329	UCUGUAAUAAAUAAUUGUAUGU
26	sli-miR-279a	UGACUAGCUCCCCCCUCAC	55	sli-miR-1814a	AGGGUUUUUGAUUUUGUUUU
27	sli-miR-34	UGACAGUGUGGACAGGUGGC	56	sli-miR-3375	GAAAAUCUUUGAAAAAUUUGGAAUA
28	sli-miR-279b	UGACGAGAUGCACUCAU	57	sli-miR-2478	UGGUGUCAGAAGUGGGAUCC
29	sli-miR-33b	GUGCAUUUGUAGUUGCAUUGCA	58	sli-miR-2507a	UUUUACGCACAAAAUUGUACAAC

**Figure 2:**
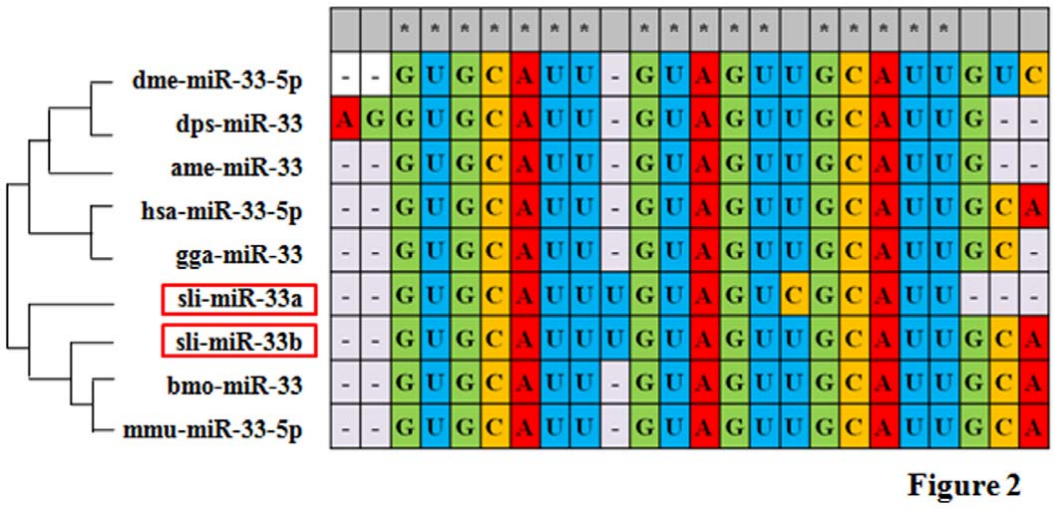
Sequence comparison and phylogenetic tree analysis of the members of the miR-33 family. The stars show the nucleotides that are conserved in all the members of the miR-33 family. sli: *S. litura*; dme: *Drosophila melanogaster*; dps: *Drosophila pseudoobscura*; ame: *Apis mellifera*; hsa: *Homo sapiens*; gga: *Gallus gallus*; bmo: *Bombyx mori*; mmu: *Mus musculus*.

Most of these 58 miRNAs have multiple homologues in different species. Over all the 58 miRNAs, the average similarity between the homologues reached 85% and some of them had similarity up to 96%, with only 1∼2 bp difference. The homologous miRNAs were generally conserved on the middle region of the sequence, but diversed at either 5′ or 3′ ends. For example, mature sli-miR-33b had 17, out of 24, nucleotides identical to its homologues in other insects, with 7 nucleotides being diverse and mainly locating at the both ends of the mature miRNAs (Fig. 2).

Analysis of the 58 miRNA revealed that they were grouped into 46 miRNA families, among which 24 are found to be exclusively present in arthropod species (miRbase), while 22 miRNA families are conserved in more than one phylum. Two miRNA families (miR-7 and miR-71) have been found in at least seven phylum, implying that they conserved in evolution and may be involved in regulation of the gene transcripts in important physiological process.

**Figure 3:**
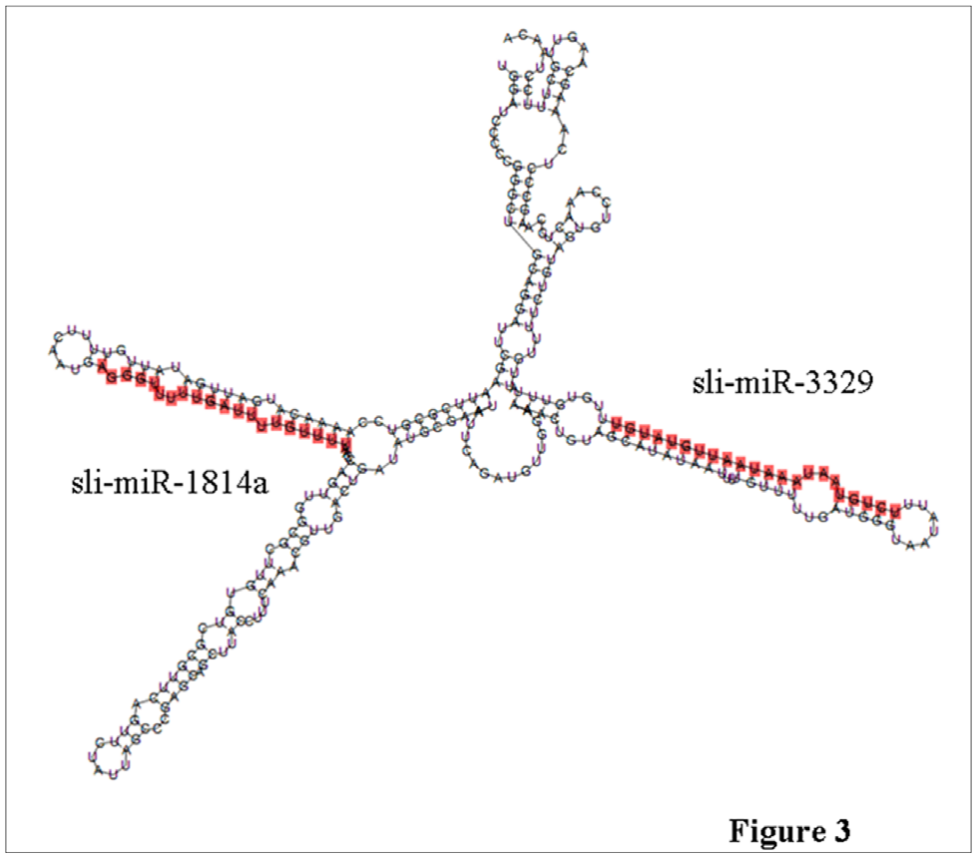
A miRNA cluster that contains sli-miR-1814a and sli-miR-3329 in the same EST isolated from *S. litura*.

One pair of the identified miRNAs sli-miR-3329 and sli-miR-1814a was found to locate in the same EST sequence with an insert of 126 nucleotides between them (Fig. 3). They are considered as clustered miRNAs [18] and may be involved in co-regulation of a same biology process.

### Expression of the Identified miRNAs

**Figure 4:**
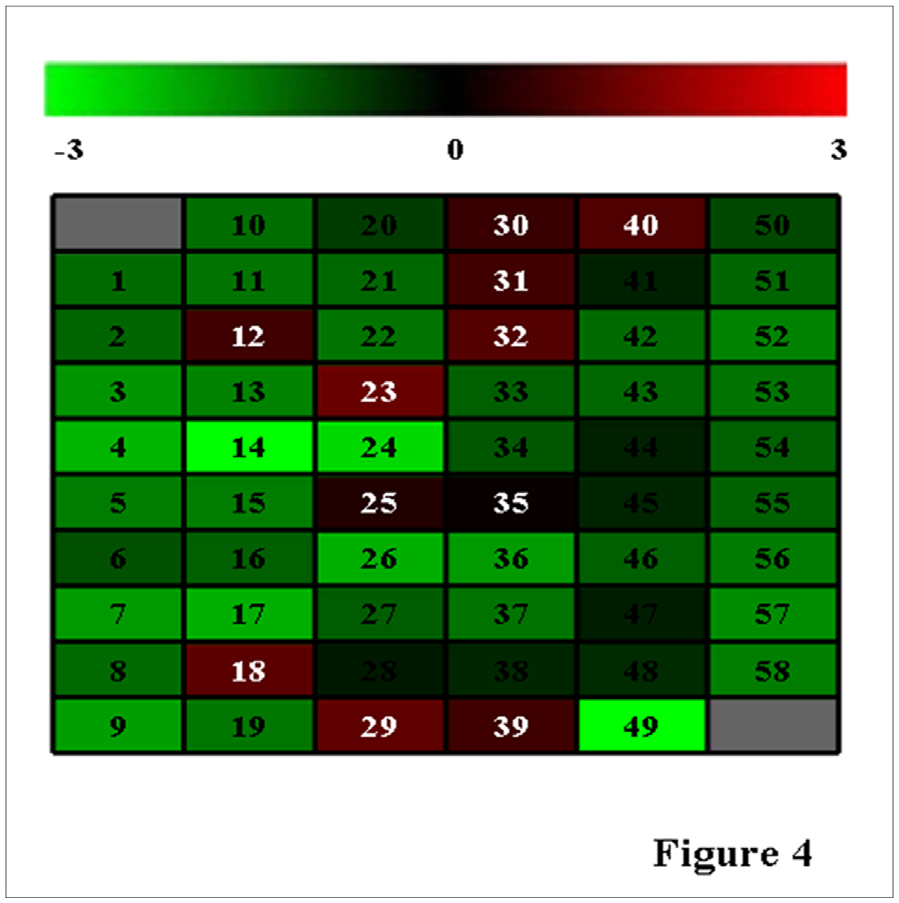
Expression of the 58 predicted miRNA detected by stem-loop RT-PCR. The expression levels of the 58 predicted miRNA of *S. litura* by stem-loop RT-PCR were digitized by ImageQuantTL after gel electrophoresis and the relative expression levels of the miRNAs were calculated as log10(miRNA/U6snRNA). The digital numbers were converted into image by using Cluster/Treeview. The scale bar shows the relative expression levels of the miRNAs over the control (U6snRNA) from −3 to 3 folds (0.001 to 1000 folds). The numbers in the figure were corresponding to those in Table 1.

To examine the expression of these 58 miRNAs, stem-loop RT-PCR were performed using a mixture of total RNA isolated from eggs, larvae, pupae and adults and using U6snRNA as control, which is believed to be highly conserved in eukaryotes [19]. The results indicated that the expression levels of 11 potential miRNAs, clones 12 (sli-miR-307), 18 (sli-miR-71), 23(sli-miR-283), 25(sli-miR-928a), 29 (sli-miR-33a), 30(sli-miR-33b), 31(sli-miR-983), 32 (sli-miR-1890), 35(sli-miR-928b), 39 (sli-miR-210) and 40(sli-miR-34), were significantly higher than the control U6snRNA (at least >2 folds) (Fig. 4). The expression levels of other 45 potential miRNAs were similar or lower than U6snRNA, with ratios being between 0.1∼1. Two miRNAs, clones 14 (sli-miR-954) and 49 (sli-miR-3001), had extremely low levels of expression with a ratio of the miRNA to the control being smaller than 0.01. The results of the stem-loop RT-PCR suggested that 56 out of 58 potential miRNAs were expressed in *S. litura* and were probably functioning miRNAs, while sli-miR-954 and sli-miR-3001 needed to be further confirmed.

### Analysis of Expression Patterns of the Selected 11 miRNAs in Different Stages

**Figure 5:**
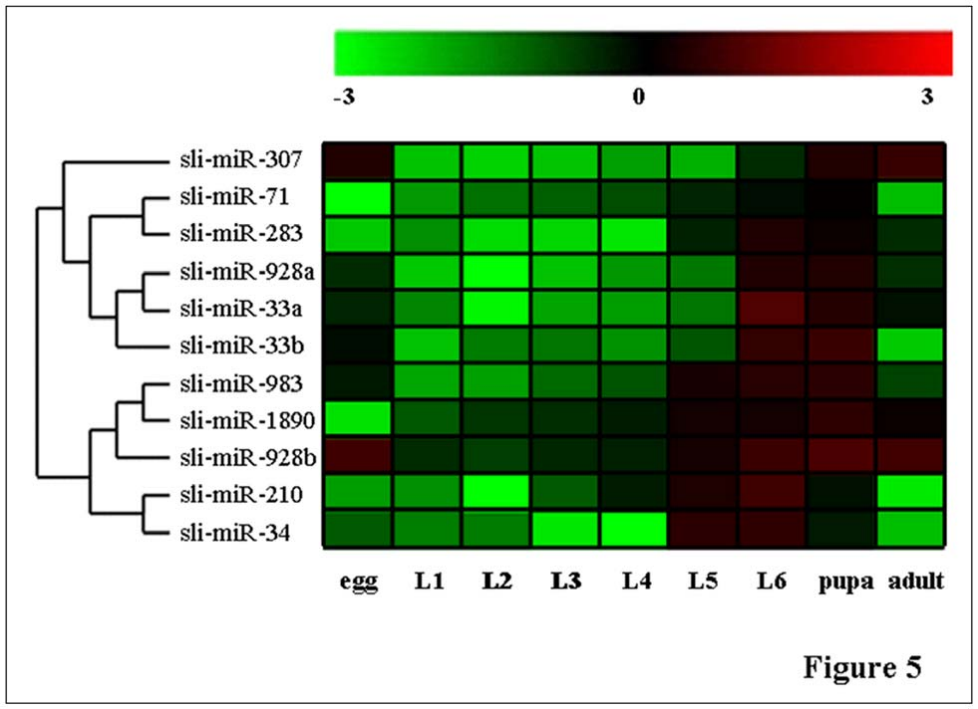
Developmental expression of the 11 selected miRNAs at different stages from eggs to adults of *S. litura*. The expression levels of the miRNA by stem-loop RT-PCR were digitized by ImageQuantTL after gel electrophoresis and the relative expression levels of the miRNAs were calculated as log10(miRNA/U6snRNA). The digital numbers were converted into image by using Cluster/Treeview. The scale bar shows the relative expression levels of the miRNAs over the control (U6snRNA) from −3 to 3 folds (0.001 to 1000 folds).

Because regulation of target mRNA transcripts by miRNA usually is a negative controlling mechanism [1], those miRNAs that are up-regulated are more interested in our study. Those 11 miRNAs that highly expressed in the insect (Fig. 4) were further analyzed for their expression pattern in eggs, larvae, pupae and adults by using stem-loop RT-PCR (Fig. 5). The results indicated that the expression of sli-miR-307 varied across the nine developmental stages. High levels of expression were detected at egg, pupal and adult stages, while low levels of expression were detected in larval stages (particularly in 2^nd^ instar) except 6^th^ instar stage. Sli-miR-71 and sli-miR-283 showed similar patterns of expression. Relative higher expression was found starting from 5^th^ instar stage to pupal stage. Sli-miR-928a and sli-miR-33a shared a very similar expression pattern with the highest levels of expression in 6^th^ instar larvae and pupae. They also have a moderate level of expression in eggs and adults. The expression of Sli-miR-33b was similar to that of sli-miR-33a and sli-miR-928a from egg to pupal stages, but no expression was detected at adult stage. The expression pattern of sli-miR-1890 was similar to that of sli-miR-928b, but had much higher levels of expression at egg and adult stages. These two miRNAs increased their expression gradually during the development from 5^th^ instar larvae to pupae. The expression pattern of sli-miR-983 was basically similar to sli-miR-928b, but sli-miR-983 had lower expression than sli-miR-928b at the egg, larval and adult stages. Sli-miR-210 and sli-miR-34 highly expressed at 5^th^ and, particularly, 6^th^ instar stages and then the expression decreased at pupal and adult stages.

In summary, all of the 11 miRNA exhibited high levels of expression during the 6^th^ instar larval and pupal stages. Sli-miR-928b, sli-miR-928a, sli-miR-307, sli-miR-983, Sli-miR-33a and Sli-miR-33b also expressed in eggs. Sli-miR-928b, sli-miR-928a, sli-miR-307, sli-miR-1890, sli-miR-283 and Sli-miR-33a expressed to different extent at adult stage. Most of the miRNAs had low levels of expression during the larval development from 1^st^ to 4^th^ instar stages.

### Analysis of Expression Patterns of the Selected 11 miRNAs in Different Tissues

**Figure 6:**
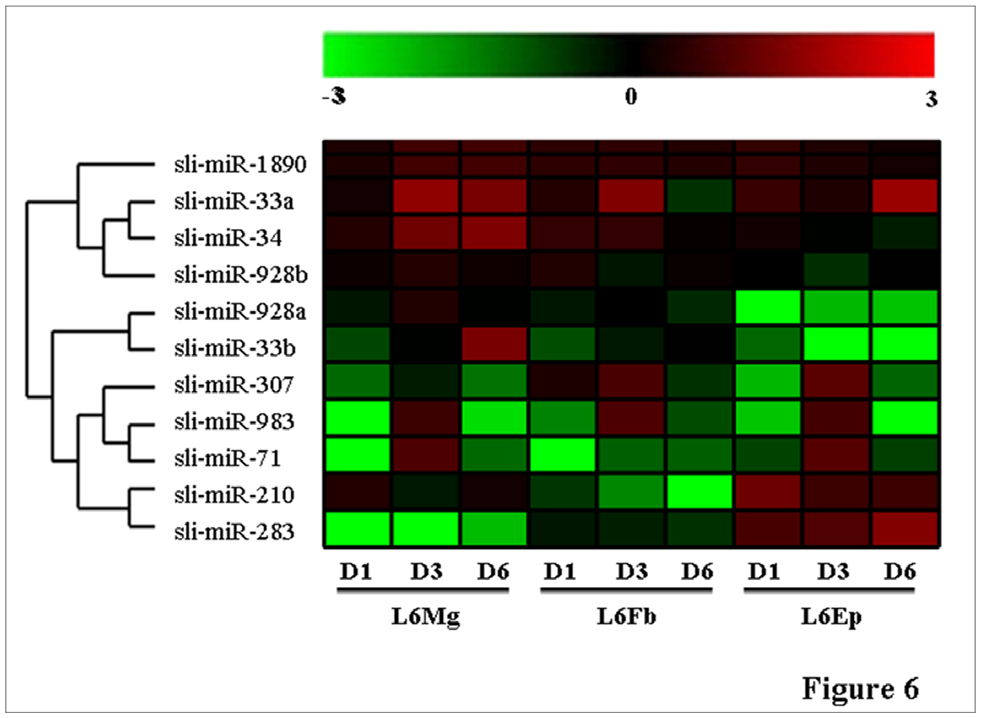
Spatial expression of the 11 selected miRNAs in the midgut, fat body and epidermis during the 6^th^ (last) instar larval stage of *S. litura*. The expression levels of the miRNA by stem-loop RT-PCR were digitized by ImageQuantTL after gel electrophoresis and the relative expression levels of the miRNAs were calculated as log10(miRNA/U6snRNA). The digital numbers were converted into image by using Cluster/Treeview. The scale bar shows the relative expression levels of the miRNAs over the control (U6snRNA) from -3 to 3 folds (0.001 to 1000 folds). Mg: midgut; Fb: fat body; Ep: epidermis. D1, D3 and D6: day 1, 3 and 6 in 6^th^ instar stage, respectively.

Because the selected 11 miRNAs highly expressed at 6^th^ instar larval and pupal stages (Fig. 5) and the original ESTs were from three stages of 6^th^ instar larvae (day 1, 3 and 6 after ecdysis into the 6^th^ instar stage), detailed analysis of expression of the miRNAs in different tissues of these three stages of 6^th^ instar larvae was performed. Total RNA was extracted from the midgut, fat body and epidermis at L6D1, L6D3 and L6D6 for stem-loop RT-PCR analysis. The results indicated that sli-miR-1890 maintained at a relatively stable and high expression level in the three tissues during the 6^th^ instar stage (Fig. 6). Sli-miR-33a and sli-miR-34 had similar expression pattern in the midgut, while Sli-miR-33a had much higher expression at L6D3 in the fat body and at L6D6 in the epidermis than sli-miR-34. sli-miR-928b basically displayed a similar expression pattern as Sli-miR-33a and sli-miR-34, but with lower levels, in different tissues at the 6^th^ instar stage. sli-miR-1890, Sli-miR-33a, sli-miR-34 and sli-miR-928b were clustered together because in general they are expressed in all the tissues of 6^th^ instar larvae. Sli-miR-928a and sli-miR-33b did not express in the epidermis, but expressed in the midgut and fat body at different levels. Sli-miR-307, sli-miR-983 and sli-miR-71 had a similar expression pattern in the midgut and epidermis, for example, they were much higher expression at L6D3 than at L6D1 and L6D6 in these two tissues. In the fat body, Sli-miR-307 and sli-miR-983 had a high expression at L6D3, while sli-miR-71 was not expressed. Sli-miR-210 and sli-miR-283 had high expression in the epidermis through 6^th^ instar larval stage, while Sli-miR-210 also expressed in the midgut and sli-miR-283 expressed in the fat body. Sli-miR-283 did not express at all in the midgut. Sli-miR-307, sli-miR-983, sli-miR-71, sli-miR-210 and sli-miR-283 were clustered into a group because they expressed in the epidermis, separating from sli-miR-928a and sli-miR-33b, which did not express in the epidermis.

In summary, sli-miR-1890, sli-miR-33a, sli-miR-34 and sli-miR-928b appeared to express in all the tissues during the 6^th^ stage, while sli-miR-928a, sli-miR-33b sli-miR-307, sli-miR-983, sli-miR-71, sli-miR-210 and sli-miR-283 were more tissue- and stage-specific in 6^th^ instar larvae.

### Target Gene Prediction of the Selected 11 miRNA

To identify potential target genes of the selected 11 miRNAs, we used the Targetscan and PicTar programs [20] to search Class I target genes and used RNAhybrid [21] to search Class II target genes. Totally, 211 potential target genes were identified for the 11 miRNAs, with 189 belonging to Class I and 22 belonging to Class II (Table 2). These target genes are involved in metamorphosis, metabolisms, apoptosis, binding activity and signal transduction.

**Table 2:** Potential target genes of the selected 11 miRNAs.

No.	miRNA	No. of target genes	Selected potential target genes
1	sli-miR-33a	2	*CG18561, CG13492*
2	sli-miR-33b	6	*CG32062, CG14006, CASK*
3	sli-miR-34	21(1)*	*Katanin60, bruchpilot, synaptotagmin, mbl*
4	sli-miR-71	14(3)	*Obstructor-E, scabrous, cnc, neurotactin*
5	sli-miR-210	27(2)	*LanB2, Khc-32, Or35a, flfl, tou, crb, pygo*
6	sli-miR-283	14(1)	*Cnc, Gr59c, mbl, Nacalpha*
7	sli-miR-307	39(2)	*Sr, fkh, Atg4, eIF-5A, kkv, LpR1, Or41a*
8	sli-miR-928a	39(6)	*R, Cip4, B-H1, smg, kuz, AP-47, Atf-2*
9	sli-miR-928b	44(7)	*R, Rab23, bur, how, AP-47, hbs, Tollo*
10	sli-miR-983	5	*CG8546, CG12996*
11	sli-miR-1890	0	-

* The numbers in the parenthese are for the second class of miRNA target genes.

### Binding of miRNA with Target Genes

**Figure 7:**
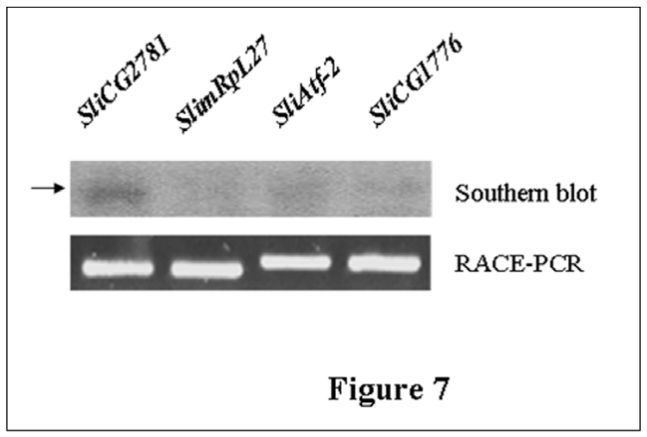
Southern blotting analysis of sli-miR-928b binding with the mRNA transcripts of its predicted potential target genes, CG2781 (GenBank accession number: 40943), mRpL27 (GenBank accession number: 318825), Atf-2 (GenBank accession number: 37978) and CG1776 (GenBank accession number: 36002). The 3′-UTR cDNA fragments that contained the sli-miR-928b complementary sequence of the four target genes were amplified by RACE-PCR (lower panel). Sli-miR-928b miRNA was synthesized and labeled with 32P-ATP as a probe and used for the hybridization with the 3′-UTR fragments of the genes (upper panel).

The identified miRNA sli-miR-928b has four predicted target genes, *CG2781*, *mRpL27*, *Atf-2* and *CG1776. CG2781* is involve in fatty acid elongation [22], [23] and *CG1776* encodes a calcium-dependent protein kinase [24], [25], [26]. *mRpL27* and *Atf-2* encode mitochondrial ribosomal protein L27 [27], [28] and activating transcription factor-2 [29], [30], respectively. To examine the possibility of sli-miR-928b binding to the transcripts of its predicted target genes, the 3′UTR cDNA fragments that contained the sli-miR-928b complementary sequence of the four target genes were amplified by RACE-PCR. Sli-miR-928b miRNA was synthesized and labeled with ^32^P-ATP as a probe. Southern blot hybridization between the 3′UTR cDNA of four target genes and the sli-miR-928b probe was performed. The results showed that *CG2781*, *Atf-2* and *CG1776* could bind with the sli-miR-928b probe in various degrees, while *imRpL27* could not (Fig. 7). The results suggested that sli-miR-928b may target the transcripts of the *CG2781*, *Atf-2* and *CG1776* genes *in vivo*.

## Discussion

### Reliability of Identified *S. litura* miRNA

Totally, 58 *S. litura* miRNAs were identified in this study. Firstly, these miRNAs were identified from 1,132 ESTs, which have no-hit homologues in the public databases, by sequence comparison with the 403 known miRNAs of insects, including *B. mori*, *A. gambiae*, *D. pseudoobscura*, *D. melanogaster* and *A. mellifera*. Some of these known insect miRNA had been demonstrated to express or function in the insects [31]. Secondly, these potential miRNAs were further confirmed by stem-loop RT-PCR. Stem-loop RT-PCR has been used to specifically amplify miRNA in many species [32], [33], [34]. In stem-loop RT-PCR the primers were designed based on specific miRNA structure, therefore, only those miRNAs that have the same nucleotide sequences as the primers can be amplified by RT-PCR. All of the 58 potential miRNA could be amplified in this study, indicating that they possessed the miRNA structure. Thirdly, three known *B. mori* miRNA (bmo-let-7,bmo-miR-7,bmo-bantan; http://miRbase.org) were used along with another three 22-bp sequences within the *B. mori* genes as negative control to test the efficiency of the stem-loop RT-PCR. The results showed that all of the three known *B. mori* miRNAs could be amplified by stem-loop RT-PCR, whereas no PCR products were amplified from the negative control. All these results together indicate that this approach used in this study can efficiently identify the potential miRNA in *S. litura*.

### Expression and Potential Function of *S. litura* miRNAs

Eleven miRNAs mainly expressed from late larval stage (L5∼L6) to pupa stage. This changing pattern in expression of miRNA is similar to another Lepidoptera insect, *Bombyx mori* [33] and fly. In fly, the expression of miRNA targets decreases starting from larval periods and lasting to adulthood, implying that the expression of miRNAs tends to be high from early larval stage (L24h) to adult [35] in fly.

Expression pattern analysis and target gene prediction can provide some clues for potential function of the *S. litura* miRNAs identified in this study. For example, miR-33 has been found to be involved in the metabolism of high density lipoprotein (HDL) and cholesterol in the pathway of fatty acid metabolism [36], [37]. In this study, sli-miR-33b was found to highly express in eggs, 6^th^ instar larvae and pupae (Fig. 5), particularly in the midgut of prepupal larvae (Fig. 6). Potential target genes for sli-miR-33b were predicted as *CG32062*, *CG2781* and *CASK*. *CG32062* encode Ataxin-2 binding protein 1 and involved in nervous system development and imaginal disc-derived wing vein specification [38]. *CG2781* is also a protein coding gene and involved in very long-chain fatty acid biosynthetic process [23]. *CASK* is involved in neurotransmitter secretion and cell shape [39], [40]. A high level of sli-miR-33b expression in eggs may imply that sli-miR-33b regulates the nervous system development. However, sli-miR-33b also had a high level of expression in the fat body of prepupal and pupal stages, implying that it may regulate the fatty acid elongation. On the other hand, although *CG32062* is predicted as a potential target gene for sli-miR-33b, but it appeared not to express in the epidermis. Whether or not this miRNA is involved in regulation of imaginal disc-derived wing vein specification is suspected.

The predicted target genes for sli-miR-307 are *sr*, *fkh, Stat92E*, *CG32467*, *Or42a* and *kkv*. *sr* is involved in central nervous system development and ectoderm development [41], [42]. *fkh* is involved in negative regulation of cell growth [43]. *Stat92E* is involved in ovarian follicle cell development [44]. *CG32467* is involved in compound eye development [45]. *Or42a* is believed to be involved in olfactory behavior and sensory perception of smell [46], [47]. *kkv* is suggested to be involved in chitin-based embryonic cuticle biosynthetic process [48], [49]. sli-miR-307 had a high level of expression in eggs (Fig. 5), implying that it may regulate the central nervous system, ectoderm development and chitin-based embryonic cuticle biosynthesis during the embryo development. Sli-miR-307 also exhibited high levels of expression at the last larval, pupal and adult stages, indicating that it may be also involved in ovarian follicle cell development, eye development and olfactory behavior.

Sli-miR-928a and sli-miR-928b had similar expression patterns and both had high levels of expression in eggs, later 6^th^ instar larvae, pupae and adults (Fig. 5). However, this similar expression pattern was detected only in the midgut and fat body, whereas their expression was different in the epidermis (Fig. 6). Sli-miR-928b expressed in the epidermis but sli-miR-928s did not. The predicted target genes common for both of sli-miR-928a and sli-miR-928b are *R* [50] and *seq* [51], [52], which regulated the cell shape and axon extension, respectively. How these miRNAs accurately and correctly regulate different processes in the tissues at various stages is interesting.

Predicted target gene of sli-miR-983 is *CG8546*, which has been suggested to have molecular function of ligand-gated sodium channel activity [53]. It may be involve in sodium ion transport by down-regulate the potential target *CG8546*. Sli-miR-283 was highly expressed in the epidermis of 6^th^ instar larvae (Fig. 6). One of its potential target genes was *mbl*, which has been demonstrated to be in involved in apoptosis [54] and compound eye photoreceptor [55]. Sli-miR-71 highly expressed in the midgut and epidermis of 6^th^ instar feeding larvae (Fig. 6). One of its possible target genes was *obstractor-E*, which has been found to be involved in chitin metabolic process [56]. Therefore, it is possible that sli-miR-71 may play a role in inhibition of *obstractor-E* and then chitin degradation in the midgut and epidermis during larval feeding stage. Similar to sli-miR-283, a potential target gene of sli-miR-34 is *mbl* that is involved in apoptosis. In fact, several studies reported that miR-34 is associated with inhibition of apoptosis [57], [58], [59].

Sli-miR-210 highly expressed in the epidermis, as well as midgut, in 6^th^ instar larvae (Fig. 6). miR-210 has been found to play an important role in endothelial migration, vascular reconstruction [60] and arteriosclerosis [61]. The predicted potential target genes of sli-miR-210 are *CG5246* and *CG6359*, which are involved in serine-type endopeptidase activity and phosphatidylinositol binding, respectively [62], [63], [64], implying that sli-miR-210 may be involved in process of proteolysis, phagocytosis and engulfment of the epidermis.

No potential target genes were predicted for sli-miR-33a and sli-miR-1890 when the 3′UTR of *D. melanogaster* was used to analysis. Sli-miR-1890 expressed in all the tissues tested in 6^th^ instar larvae (Fig. 6). Sli-miR-33a was the most highly expressed miRNA among all of the identified miRNAs and it expressed in all the tissues, particularly in the midgut of 3-day-old 6^th^ instar larvae and prepupal larvae (day 6 of 6^th^ instar stage) and in the epidermis of prepupal larvae. Because these two miRNAs always have high levels expression, it is probably they suppress some genes that are not benefic to cellular functions or activities.

Eleven potential TFs (*CG32062, mbl, cnc, CG11776, CG15455, CG34209, fkh, pdm3, CG14478, Atf-2, His4:CG33907*) were found among the 128 predicted target genes, being about 8.5% of the all target genes identified. The ratio of TFs to nuclear proteins in human is 50% (19/38) and to all the target genes in cellular signaling networks is 11.9% (19/159) [65]. Based on the search in the MicroCosm (http://www.ebi.ac.uk/enright-srv/microcosm/cgi-bin/targets/v5/search.pl), the ratio of all the TFs and co-factors to all the target genes in human is 6.7% (2363/34788). In *D. melanogaster* this number is 7.2% (869/12046); in *A. gambiae* it is 6.2% (606/9721); in *Mus musculus* it is 7.6% (2316/30484). These data indicate that the TFs apparently account for less than 10% of the total miRNA target genes in these species.

## Materials and Methods

### Experimental Insects

Second instar larvae of *Spodoptera litura* was provided by The Entomology Institute of Sun Yat-Sen University, Guangzhou, China, and reared at 27±1°C a relative humidity of 65%∼75% in the laboratory until adults. The larvae at the selected stages were placed on ice and carefully dissected to isolate different tissues such as midgut, epidermis and fat body for RNA extraction, or immediately put into liquid nitrogen and stored at −80°C until use.

### EST Dataset Generation

Total RNA were extracted using Trizol Reagent (Gibco, USA) and mRNAs were isolated using Oligotex mRNA Kits (Qiagen, USA) from the midguts of the 5^th^ to 6^th^ instar molting larvae (day 1 in 6^th^ instar, L6D0), 6^th^ instar feeding larvae (day 3 in 6^th^ instar, L6D3) and prepupae (day 6 in 6^th^ instar, L6D6). cDNA libraries were constructed using pBluescript II KS (+) cDNA Library Construction Kit (Stratagene, USA). Bacterial clones were randomly selected from the three cDNA libraries and subjected to 5′-single pass sequencing using Applied Biosystems (ABI) 3730 DNA sequencer, generating 6,827 high-quality ESTs (Qili Feng et al., data not published). The ESTs were assembled and blast searched for homologues in the public databases such as nr, nt and dbEST databases of NCBI GenBank (http://www.ncbi.nlm.nih.gov/genbank/), InterPro (http://www.ebi.ac.uk/Tools/pfa/iprscan/) and ExPASy (http://expasy.org/proteomics/protein_sequences_and_identification).

### Computational Prediction

A pipeline was set up for homologue searching and identification of miRNA from expressed sequence tags of *S. litura* (Fig. 1). Firstly, 403 known insect miRNAs in the miRbase were used to blast the annotated 1,132 ESTs that were obtained from the midgut cDNA libraries of *S. litura* and do not contain complete open reading frames (Qili Feng et al., unpublished data). The EST sequences that had less than 5 mismatching nucleotides against the known miRNAs were regarded as the homologous sequences of the corresponding miRNAs and the franking regions plus the matching nucleotide sequences were taken as the potential miRNA precursors. Analysis of secondary structure and free energy of these sequences was conducted by using the RNAfold/Mfold program [66], [67]. A threshold value of free energy was set at ΔG≤−20 kcal/mol. Classical stem-loop structure, core mfe, Ch_ratio, GC content and mismatch nucleotides were also set up and used as criterions for identification of putative miRNAs [33]. Only those sequences that satisfied all these criterions would be considered as a potential miRNA. If more than one precursor agrees with those criterions for a potential miRNA, the one with lowest free energy was considered as a possible precursor.

### Total RNA Extraction and Small RNA Enrichment

Total RNAs were extracted using RNAiso Plus (TAKARA) according the manufacturer’s instruction from eggs, 1^st^ instar to 6^th^ instar larvae, pupae and adults, respectively. The quality of total RNAs was examined by spectrum analysis using Nanodrop ND-100 Spectrophotometer (GE Healthcare Life Sciences, Piscataway, USA) and gel electrophoresis.

Small RNAs (less than 200 nt) were enriched by PEG 6000 centrifugation (in 13% NaCl). The size range of small RNAs was examined by electrophoresis in polyacrylamide gel.

### Synthesis of cDNA and PCR Analysis of miRNAs

cDNA was synthesized from the small RNAs by using miRNA specific stem-loop primers, which were obtained from Invitrogen (California, USA). The mix of RNA template and primers contained 150 ng small RNA sample, 0.5 µl stem-loop RT primer (10 mM), 0.5 µl dNTPs (10 mM) and RNase-free ddH_2_O up to 7 µl. The mixture was degenerated at 65°C for 5 min and immediately cooled down on ice in 2 min. Reverse transcriptase reactions contained 7 µl of the template/primer mix, 2.5 U Reverse Transcriptase XL (AMV), 2 µl 5×AMV buffer, 20 U RNase inhibitor in a total of 10 µl. The reactions were carried out at 16°C for 30 min; then follow by 60 cycles at 20°C for 30 sec, at 42°C for 30 sec, at 50°C for 2 sec; at 85°C for 10 min and then hold at −20°C. The synthesized cDNAs were used for PCR analysis of miRNA expression or stored at −80°C for use later.

For PCR analysis, the PCR mixtures contained 1 µl cDNA without dilution, 2 U Taq (5 U/µl), 3.6 µl 10×PCR buffer (10 mM), 1 µl dNTPs (10 mM), 0.8 µl F-primer (10 mM), 0.8 µl URP (10 mM) in a total volume of 20 µl. The PCR reaction were performed in 200 µl micro-tube at 95°C for 10 min; at 55°C for 2 min; followed by 30 cycles at 95°C for 1 sec, 65°C for 1 min, and finally at 72°C for 5 min. The PCR products were analyzed by electrophoresis in 3% agarose gel containing ethidium bromide (0.5 µg/µl) and photographed using ImagaQuant 300 (GE Healthcare Life Sciences, Piscataway, USA).

### Cloning of *S. litura* miRNA

After electrophoresis, PCR products were recovered from the gels according to Gel DNA Fragment Recovery Kit V 2.0 (Axygen, Hangzhou, China). The recovered cDNA products were ligated to pMD-18T vector (TaKaRa Co., Dalian, China) using T4 ligase and the recombinant DNA vectors were used to transfect *Escherichia coli* DH5α competent cells. Positive clones were selected and confirmed by PCR reaction (0.1 µl Taq, 0.2 µl dNTPs, 1 µl 10× PCR buffer, 0.2 µl F-primer, 0.2 µl URP, add ddH_2_O up to 15 µl). The reaction conditions were 94°C for 3 min; the follow by 30 cycles of 94°C for 30 sec, 55°C for 30 sec, 72°C for 1 min and finally 72°C for 10 min. DNA of the positive clones was then extracted and sequenced.

### Prediction of Target Genes for miRNAs

For predicting miRNA target genes, 3′UTR sequences of *Drosophila melanogaster* genes from the unigene database in NCBI were subjected to predict the first class of target genes by using the Targetscan(http://www.targetscan.org/fly_12/) and PicTar programs [20] (http://pictar.mdc-berlin.de/) with default parameters at threshold value of free energy ΔG<−20 kcal/mol. Fragments of 12^th^ nt∼ (x-2)^th^ nt (x is the total length of the selected miRNA) of miRNAs were also used to blast the 3′UTR of *D. melanogaster* genes using RNAhybrid [21] (http://bibiserv.techfak.uni-bielefeld.de/) to determine the free energy. When the free energy ΔG between two molecules was smaller than −20 kcal/mol, the genes were considered as potential target genes for the corresponding miRNAs.

Southern blot analysis was performed to examine binding of identified miRNAs to the predicted potential target genes. Total RNA was extracted from the larval midgut at days 1, 2 and 3 of 6^th^ instar stage and then mixed together. The 3′UTR fragments of the predicted target genes were cloned from *S. litura* using primers designed based on the target gene sequences and by RACE-PCR according to SMART RACE cDNA Amplification Kit protocol (Clontech, California, USA). The amplified DNA was sequenced for confirmation. The DNA was then detected by electrophoresis on 1% agarose gel and then transferred to Amersham Hybond™-N^+^ membrane. DNA Probes complementary to the miRNA were synthesized and labeled with [γ-^32^P] ATP. Membrane was pre-hybridized in prehybridization solution containing 6×SCC, 10×Denhardt’s solution and 0.2% SDS at 65°C, 3 rpm for at least 4 h. The membrane was then hybridized in hybridization solution containing 6×SSC, 5×Denhardt’s solution, 0.2%SDS with 1∼5×10^6^ cpm [γ-^32^P] ATP -labeled probe at 42°C, 3 rpm for about 24 h. The membrane was washed three times for 5 min each at room temperature with 6×SSC and 0.2%SDS and then washed once with 6×SSC and 0.2%SDS at 42°C for 15 min. After final wash, the membrane was exposed to phosphorus screen for about 48 h and the signal was detected using a Typhoon Trto Vanriable Mode Imager (Amersham, Piscataway, USA).
